# Attitudes towards gender roles and prevalence of intimate partner violence perpetrated against pregnant and postnatal women: Differences between women immigrants from conflict-affected countries and women born in Australia

**DOI:** 10.1371/journal.pone.0255105

**Published:** 2021-07-30

**Authors:** Madelyn Hsiao-Rei Hicks, Mohammed Mohsin, Derrick Silove, Jane Fisher, Batool Moussa, Zachary Steel, Heather Nancarrow, Nawal Nadar, Louis Klein, Fatima Hasoun, Mariam Yousif, Batoul Khalil, Yalini Krishna, Susan J. Rees

**Affiliations:** 1 Department of Psychiatry, University of Massachusetts Medical School, Worcester, Massachusetts, United States of America; 2 School of Psychiatry, Faculty of Medicine, University of New South Wales, Kensington, New South Wales, Australia; 3 Mental Health Academic Unit, Liverpool Hospital, South Western Sydney Area Health Service, New South Wales, Australia; 4 Women and Global Health Unit, School of Public Health and Preventive Medicine, Monash University, Melbourne, Victoria, Australia; 5 St John of God Health Care, Richmond Hospital, North Richmond, New South Wales, Australia; 6 School of Social Sciences, Arts and Social Sciences, University of New South Wales, Kensington, New South Wales, Australia; University of Salamanca, SPAIN

## Abstract

**Background:**

The aim was to compare, for the first time in a large systematic study, women born in conflict-affected countries who immigrated to Australia with women born in Australia for attitudes towards gender roles and men’s use of IPV and the actual prevalence of IPV. The study also examined if any associations remained across the two timepoints of pregnancy and postpartum.

**Methods:**

Women were interviewed during their first visit to one of three Australian public hospital antenatal clinics and re-interviewed at home six months after giving birth. A total of 1111 women completed both interviews, 583 were born in conflict-affected countries and 528 born in Australia. Associations between attitudes towards gender roles and men’s use of IPV, socio-demographic characteristics and reported actual experiences of IPV were examined using bivariate and multiple logistic regression analyses.

**Results:**

Attitudes toward inequitable gender roles including those that condone men’s use of IPV, and prevalence of IPV, were significantly higher (*p*<0.001) among women born in conflict-affected countries compared to Australia-born women. Women born in conflict-affected countries with the strongest held attitudes towards gender roles and men’s use of IPV had an adjusted odds ratio (aOR) of 3.18 for IPV at baseline (95% CI 1.85–5.47) and an aOR of 1.83 for IPV at follow-up (95% CI 1.11–3.01). Women born in Australia with the strongest held attitudes towards gender roles and IPV had an aOR of 7.12 for IPV at baseline (95% CI 2.12–23.92) and an aOR of 10.59 for IPV at follow-up (95% CI 2.21–50.75).

**Conclusions:**

Our results underscore the need for IPV prevention strategies sensitively targeted to communities from conflict-affected countries, and for awareness among clinicians of gender role attitudes that may condone men’s use of IPV, and the associated risk of IPV. The study supports the need for culturally informed national strategies to promote gender equality and to challenge practices and attitudes that condone men’s violence in spousal relationships.

## Introduction

Intimate partner violence (IPV) against women remains a highly prevalent problem worldwide with immediate and long-term negative impacts on women’s mental, physical, economic, and social wellbeing [1–11]. Mental health problems, in which anxiety and depression predominate, are twice as likely to be reported by women who have experienced intimate partner violence compared to those who have not, with a higher risk of associated poor health [4–6]. Women from conflict-affected settings who have resettled in high-income countries may be at increased risk of IPV because of the compounding impacts of conflict and of displacement-related trauma, as well as greater social and economic disadvantages [12]. Moreover, women from refugee backgrounds who experience IPV are particularly vulnerable to mental health problems during pregnancy [12]. Although IPV is a risk factor for poor health outcomes in medical and public health terms, it is important to recognize that IPV is also a violation committed by a perpetrator, predominantly towards women, which breaches women’s human rights. Given the extensive health, social, and human rights impact of IPV, identifying potentially modifiable contributors is a priority [1–3, 6, 8–11].

Attitudes that support gender inequality and violence towards women have been found to be amenable to relatively rapid change [13]. Nevertheless, IPV prevention campaigns in high-income countries have not prioritized nor specifically focused on attitudes towards gender roles and IPV among populations coming from conflict-affected countries, particularly people who are refugees or who have experienced humanitarian crises. A reason for this oversight may be that research on associations between attitudes towards gender roles and IPV and prevalence of IPV have been largely limited to stable populations unaffected by mass conflict. [3, 5, 14].

In general populations, men’s attitudes toward masculinity, male entitlement to control women, and women’s secondary role in relationships have been found to be associated with the prevalence and perpetration of IPV [15]. Women who hold gender role values supportive of IPV and traditional gender role values may be more likely to blame themselves for an assault, and less likely to report IPV to police or other authorities [15, 16]. Research on associations between IPV and attitudes towards gender roles and IPV in conflict-affected populations is especially important because rates of IPV and associated health problems are elevated in women exposed to and displaced by armed conflict and political violence [17], where war-related trauma is viewed as a significant factor in explaining violence within the family [9, 18–21]. Research is needed to examine the possible links between IPV prevalence and gender role and IPV attitudes in the high-risk group of women who resettle in high-income countries after fleeing conflict in their home countries. In this group IPV prevalence may be high and trauma, loss of trust in authorities, social isolation, and cultural differences may further reduce their capacity to seek support or to escape IPV [22]. In addition to being in a high-risk category for IPV, absolute numbers of women who have experienced armed conflict and displacement are substantial in high-income countries, with a heightened risk for mental health problems during settlement [12].

Studies of associations between attitudes towards gender roles and IPV and prevalence of IPV in conflict-affected populations are limited, with only two empirical studies identified at the time of writing. In the first study, a household survey of 262 women and 133 men in 12 Palestinian refugee camps in Jordan found that women who had experienced IPV were significantly more likely to have attitudes that condoned wife-beating than women who had not experienced IPV, and men with attitudes that condoned wife-beating were significantly more likely to have perpetrated IPV [23]. In the second study, which used sequential sampling of 409 women attending two health clinics in northern Uganda where the population is severely affected by conflict, no association was found between experiences of IPV and attitudes that justified wife-beating [24]. Neither study applies directly to settings of high-income countries of resettlement where norms and conditions may be different for women because of the absence of current internecine conflict, and the presence of legislated protections and cultural standards reflecting women’s rights to safety and autonomy.

Despite the recognized importance of ensuring that often traumatized conflict-affected women living in high-income countries are safe from violence and adequately supported during settlement, only one small study was found to be relevant to the present inquiry. The study used snowball sampling to compare 21 Bosnian refugee women living in the United States with 49 non-refugee Bosnian women living in the home country of Bosnia-Herzegovina. Similar rates of experienced IPV and of attitudes condoning IPV were found across the two groups, however the study did not look for associations between IPV and IPV attitudes [25].

This is a novel study of associations between IPV and attitudes towards gender roles and men’s use of IPV amongst women born in conflict-affected countries during their pregnancy and in the postnatal period. The main aim of the study was to compare women born in conflict-affected countries with women born in Australia in regard to their attitudes to gender roles and IPV and prevalence of the actual experience of IPV. We also aimed to study associations between their IPV exposure, socio-demographic characteristics and attitudes towards gender roles and IPV. We also examined the stability of these outcomes across pregnancy and the postnatal period. Pregnancy and postnatal periods were chosen as times of particular importance because of higher rates of IPV during pregnancy found in some studies, and the higher risk of IPV for pregnant women of minority status. The period of routine perinatal care, both during pregnancy and postnatal care also offers the opportunity for screening, interventions, and data collection on IPV [26]. The study hypothesis was that woman born in conflict-affected countries who immigrated to Australia would have attitudes more supportive of gender roles and IPV, and higher rates of IPV, than women born in Australia.

## Methods

### Ethics and research personnel

The study was approved by the Southwestern Sydney Local Health District Human Research Ethics Committee (HC13049) and Monash Health Ethics Committee. Participants were provided with written and verbal information about the study, and those electing to participate signed written consent forms. No minors were included in this study.

Eight women field workers from matching language backgrounds were given extensive training consisting of three formal training days followed by tests of competence. Training covered intimate partner violence, research methods and practice, sensitive interviewing techniques, and use of the diagnostic and World Health Organization (WHO) measures. Staff received ongoing support, monitoring and supervision during the study. Inter-rater reliability tests were conducted serially to maintain standards, based on group observations of videotaped interviews. WHO guidelines were strictly adhered to for conducting safe and ethical IPV research [27].

### Participants and recruitment

Participants were recruited between January 2015 and March 2016. The recruitment of participants born in Australia and of participants from conflict-affected countries occurred at the same time and from the same three public antenatal clinics; two in the city of Sydney, New South Wales, Australia, and one in the city of Melbourne, Victoria, Australia, as part of the WATCH (Women Aware Together with their CHildren) prospective cohort study [12]. The three study sites were selected due to their positioning within areas known to have substantial populations of immigrants from conflict-affected regions [12]. The recruitment of women from conflict-affected countries included all conflict-affected Arabic-speaking countries, Sudan, and Sri Lanka (Tamil-speaking). These groups represented the largest populations immigrating from conflict-affected countries to Australia and to other high-income countries at the time of the study [12]. The study included only these predominant language groups to minimize problems of transcultural measurement error and small cell sizes. Country of origin was identified by clinic records, requests for an interpreter, or culturally recognisable surnames, and country of birth data were checked against clinic appointment lists [12]. A widely used systematic approach was applied to infer ethnicity from culturally recognizable surnames [28]. Lists were generated from the participating hospital antenatal clinics’ first appointment register, after which each name was scrutinized by experts from those cultural and language backgrounds to identify potential participants [12].

Consecutive sampling was used to systematically recruit women from conflict-affected countries. Women born in Australia attended the clinics in substantially larger numbers than those from conflict-affected countries. To undertake a parallel sampling strategy over a similar time frame, we applied a computer-generated randomisation procedure to identify daily a subset of women born in Australia. Exclusion criteria applied to women with overt psychosis, severe medical illness, and obvious intellectual impairment.

Recruitment and the baseline interview occurred at a woman’s first appointment at the antenatal clinic, which most occurred between 12- and 20-weeks’ gestation of pregnancy (range, 9–42 weeks). Women members of the research team who were fluent in the same language as eligible women approached potential participants in the waiting room and, following consent, conducted interviews lasting a maximum of 1 hour in a private area of the clinic, with breaks for refreshments or to attend to children. Follow-up interviews were conducted with women at home either by telephone or in person approximately 6 months after the birth of the index child. The rationale for interviewing women at these two points was in time twofold. First, in general, data collected longitudinally can strengthen the robustness of prevalence data, including for IPV and for the assessment of attitudes. Second, collecting data during pregnancy and then 6 months after the delivery of the index child could reveal shifts in reporting negative experiences or attitudes, especially during pregnancy, a time known to elicit a greater reluctance to report IPV [26]. Despite any hesitancy to report, pregnancy has also been associated with higher rates of IPV, even when compared with the postpartum period, and interviewing at these two points in time would allow a comparison to be made [29].

### Survey measures

#### Cultural accuracy

All instruments were selected based on their previous psychometric evaluations and use across cultures. Translations of instruments were subjected to systematic monitoring of cultural and linguistic accuracy in the study’s languages using the Translation Monitoring form approach [30]. After translation and back-translation procedures were performed, final refinements were made by groups of linguistic experts.

#### Socio-demographic characteristics

The Australian National Census items were adopted to assess place of usual residence, age, marital status, highest level of educational attainment, household composition, and employment [12].

#### Attitudes towards gender roles and IPV

Attitudes towards gender roles and IPV were measured using ‘Attitudes Towards Gender Roles’ items from the WHO Multi-Country Study on Women’s Health and Life Experiences Questionnaire [31, 32]. Attitudes were measured by 16 items in three domains presented in Box 1.

**Box 1. Gender attitude items for domains A, B and C**

**Domain A** measured attitudes towards women’s and men’s rights and roles and consisted of six items:

1. A good wife obeys her husband even if she disagrees;

2. Family problems should only be discussed with people in the family;

3. It is important for a man to show his wife/partner who is the boss;

4. A woman should be able to choose her own friends even if her husband disapproves;

5. It’s a wife’s obligation to have sex with her husband even if she doesn’t feel like it;

6. If a man mistreats his wife, others outside of the family should intervene.

**Domain B** measured attitudes towards physical violence by the male partner and consisted of six items asking, ‘In your opinion, does a man have a good reason to hit his wife if’:

7. She does not complete her household work to his satisfaction;

8. She disobeys him;

9. She refuses to have sexual relations with him;

10. She asks him whether he has other girlfriends;

11. He suspects that she is unfaithful;

12. He finds out that she has been unfaithful.

**Domain C** measured attitudes about sexual rights and sexual IPV and consisted of four items asking, ‘In your opinion, can a married woman refuse to have sex with her husband if’:

13. She doesn’t want to;

14. He is drunk;

15. She is sick;

16. He mistreats her.

For Domains A and B, items were coded 1 for ‘Agree’ and 0 for ‘Disagree’. It is to be noted that in the questionnaire, items belonging to ‘Domain C’ are phrased in reversed, negative manner. To be consistent with ‘Domain A’ and ‘Domain B’ for the statistical analysis, items in ‘Domain C’ were recoded as 0 for ‘Agree’ and 1 for ‘Disagree’. In keeping with conventions of the field, total counts of ‘Agreed’ were generated by adding all the items for each domain, respectively. Because of skewed distributions for total counts within individual domains as well as consideration of associations with IPV, each domain count was grouped into one of two categories (Domain A: 0 = 0–2 and 1 = 3 or more; Domains B and C: 0 = 0 and 1 = 1 or more). Initial analysis showed that the three domains of gender attitudes were significantly correlated. Based on dichotomous values of 0 or 1 in each of three domains, a composite attitudes towards gender roles and IPV index was created in which values could range from 0 to 3 (0 = disagreement for all three domains, 1 = agreed in one domain, 2 = agreed in two domains, 3 = agreed in all three domains). All the analyses in this paper used the composite index, which is referred to as the ‘attitudes towards gender roles and IPV index’. Mean scores and exact scores were used in the statistical analysis (0, 1, 2, 3).

#### Intimate partner violence

Intimate partner violence was assessed using items from the World Health Organization (WHO) Violence Against Women questionnaire which enquires into physical, psychological and sexual violence [3, 31–33]. Questions were asked about IPV perpetrated by the last or current partner during the past 12 months preceding the interview date. Changes in reported IPV between baseline and follow-up interviews could be due to new onset of IPV since the baseline interview, changes in willingness to report, or changes in recall. The WHO measure was applied in the Multi-Country Study on Women’s Health and Domestic Violence across 14 countries globally [3]. Based on the advice of cultural experts, explicit sexual abuse items were not included. Women were initially assigned to one of three hierarchically ordered categories: 1) No IPV; 2) Psychological IPV (without physical abuse, including jealous or angry if she talks to other men, frequent accusations of being unfaithful, does not permit meetings with female friends, limits contact with family, insists on knowing woman’s whereabouts, humiliates her in front of others, threatens harm to her or someone close to her), or 3) Physical IPV with or without psychological IPV (any physical abuse including pushing, shaking, throwing items, slapping, twisting arm; punching, kicking, dragging, strangling, burning, threats with a knife, gun or other weapon, attacks with a knife, gun or other weapon). Women were then assigned into one of two IPV categories: 1. No IPV or, 2. Any IPV (psychological IPV or physical IPV).

### Statistical analyses

Descriptive statistics were calculated for key sociodemographic variables including age, highest level of education attained, and employment status, for attitudes towards gender roles and IPV, and for IPV in Australia-born women and in women from conflict-affected countries. Bivariate analysis was used to examine for associations between sociodemographic characteristics and outcome variables of attitudes towards gender roles and IPV and IPV in Australia-born women and in women from conflict-affected countries at baseline and at follow-up. Chi-square (χ^2^), *t*, and *F* tests were used to investigate differences between target subgroups with the alpha threshold set at *p* <0.05 for two-tailed tests. As the main outcome variable, IPV measured at baseline and follow-up, was addressed as a dichotomous dependent variable (no IPV or any IPV), multiple logistic regression analyses were used to examine the relative contributions of each potential explanatory variable to the to the likelihood of any IPV model [34]. Four separate multiple logistic regression analyses were performed, two on each group of women, to estimate the relative contributions of attitudes towards gender roles and IPV to IPV at both baseline and at follow-up adjusting for socio-demographic factors in the model [34]. Adjusted odds ratios (aORs) with 95% confidence intervals (95% CI) are provided. Statistical analyses were performed by using IBM SPSS version 26. Data sufficient to replicate the study are provided in S1 Data.

## Results

### Sample characteristics

Of 1574 eligible pregnant women recruited at the time of first appointment in the antenatal clinic, which most occurred between 12- and 20-weeks’ gestation, 1335 completed baseline interviews (84.8% response rate) of which 650 (48.7%) were Australia-born and 685 (51.3%) were born in conflict-affected countries. The follow-up interview was conducted at home either in person or by telephone approximately six months after the index child was born. Women who did not participate in the follow-up interview were excluded from the study and analysis.

A total of 1111 women 83.2% of 1335) were included in this study, of whom 528 (47.2%) were Australia-born and 583 (52.5%) were born in conflict-affected countries. The most common reason for non-participation in follow-up was being uninterested in the study, followed by being too busy, feeling unwell, presence of the index child, and hostility by partners or relatives. Women born in conflict-affected countries were predominantly from Iraq (37.7%, 220 of 583), Lebanon (17.5%, 102 of 583), Sri Lanka (11.1%, 65 of 583) and Sudan (9.1%, 53 of 583). The mean age was 29.1 years (SD 5.4) for Australia-born women and 29.8 years (SD 5.4) for women from conflict-affected countries. Of Australia-born women, 42.6% (225 of 528) had no post-school qualifications and 31.6% (167 of 528) held university degrees. Of women born in conflict-affected countries, 49.2% (287 of 583) had no post-school qualification and 33.8% (197 of 583) held university degrees. Unemployment was reported by 40% (211 out of 528) of Australia-born women and by almost three-quarters (73.2%, 427 of 583) of women born in conflict-affected countries (Table 1).

**Table 1:** Descriptive statistics for sociodemographic characteristics, attitudes towards gender roles and IPV index, and intimate partner violence (IPV) for Australia-born women compared to women born in conflict-affected countries at baseline and at follow-up.

Sample characteristics	Australia-born women (n = 528)		Women born in conflict-affected countries (n = 583)		Australia-born vs. Women born in conflict-affected countries
	Number	%	Number	%	*p*-values
**Age group (in years)**					
<25	115	21.8	102	17.5	
25–34	321	60.8	362	62.1	
35 and above	92	17.4	119	20.4	
*Mean (SD)*	*29*.*1 (5*.*4)*		*29*.*8 (5*.*4)*		***0*.*039***
**Highest level of educational attainment**					
No post-school qualification	225	42.6	287	49.2	
Diploma or vocational education	136	25.8	99	17.0	
University degree	167	31.6	197	33.8	***0*.*001***
**Employment status**					
Unemployed	211	40.0	427	73.2	
Employed	317	60.0	156	26.8	***0*.*001***
**Attitudes towards gender roles and IPV index at baseline**					
0 (disagreement for all three domains)	421	79.7	253	43.4	
1 (agreed in one domain)	92	17.4	243	41.7	
2 (agreed in two domains)	14	2.7	73	12.5	
3 (agreed in all three one domains)	1	0.2	14	2.4	
***Mean (SD)***	***0*.*2 (0*.*5)***		***0*.*7 (0*.*8)***		***0*.*001***
**Attitudes towards gender roles and IPV index at follow-up**					
0 (disagreement for all three domains)	439	83.1	284	48.7	
1 (agreed in one domain)	77	14.6	204	35.0	
2 (agreed in two domains)	10	1.9	73	12.5	
3 (agreed in all three domains)	2	0.4	22	3.8	
***Mean (SD)***	***0*.*2 (0*.*5)***		***0*.*7 (0*.*8)***		***0*.*001***
**Intimate partner violence at baseline**					
No IPV	391	74.1	330	56.6	
Any IPV	137	25.9	253	43.4	***0*.*001***
**Intimate partner violence at follow-up**					
No IPV	385	72.9	327	56.1	
Any IPV	143	27.1	256	43.9	***0*.*001***

### Attitudes towards gender roles and IPV

Means for both baseline and follow-up attitudes towards gender roles and IPV indices were significantly higher in women born in conflict-affected countries (0.7, SD 0.8) compared to Australia-born women (0.2, SD 0.5, *p*<0.001) (Table 1). More than half of women born in conflict-affected countries had attitudes towards gender roles and IPV indices of 1 or more (overall agreement for one or more domains) at both baseline (56.6%) and follow-up (51.3%). Attitudes towards gender roles and IPV indices of 1 or more in Australia-born women occurred in 20.3% at baseline and 16.9% at follow-up. The higher gender roles and IPV attitudes index of 2 or 3 was significantly more prevalent in women born in conflict-affected countries at baseline (14.9%) and follow-up (16.3%) than in Australia-born women at baseline (2.9%) and follow-up (2.3%) (Table 1).

Irrespective of country of birth, women’s attitudes towards gender roles and IPV at follow-up were correlated significantly with their attitudes towards gender roles and IPV at baseline (Australia-born women: r = 0.50, *p*<0.001; Women born in conflict-affected countries: r = 0.47, *p*<0.001). In a previous analysis we found that time since arrival was not significantly associated with any of the outcome measures and did not show any specific pattern of relationship. The slight decline in attitudes towards gender roles and IPV indices in each group over time was not statistically significant (Table 2).

**Table 2:** Associations of baseline and follow-up attitudes towards gender roles and IPV indices with intimate partner violence (IPV) in Australia-born women and in women born in conflict-affected countries.

Australia-born women and Women born in conflict-affected countries by attitudes and IPV	Baseline	Follow-up	Statistical differences between baseline and follow-up; *p*-value	Correlation co-efficient (r) between baseline and follow-up; *p*-value
	***Attitudes towards gender roles and IPV index*: *mean (95% CI)***			
Australia-born women (n = 528)	0.23 (0.19–0.28)	0.19 (0.16–0.24)	*p* = 0.070; Paired samples t-test	r = 0.502; *p*<0.001
Women born in conflict-affected countries (n = 583)	0.74 (0.68–0.80)	0.71 (0.65–0.78)	*p* = 0.449; Paired samples t-test	r = 0.473; *p*<0.001
*p-values*: *Australia-born vs*. *Born in conflict-affected countries*	*p*<0.001	*p*<0.001		
	**Prevalence of IPV: n; % (95% CI)**			
Australia-born women (n = 528)	n = 137; 25.9% (22.2–29.7)	n = 143; 27.1% (23.3–30.9)	*p* = 0.637; McNemar’s test	r = 0.456; *p*<0.001
Women born in conflict-affected countries (n = 583)	n = 253; 43.4% (39.4–47.4)	n = 256; 43.9% (39.9–47.9)	*p* = 0.612; McNemar’s test	r = 0.522; *p*<0.001
*p-values*: *Australia-born vs*. *Born in conflict-affected countries*	*p*<0.001	*p*<0.001		

In the bivariate analysis, higher attitudes score on the gender roles and IPV index was significantly correlated with unemployment and with reported IPV in both Australia-born women and in women born in conflict-affected countries. The association was found both at baseline and at follow-up. For women born in conflict-affected countries, having no post-school educational qualifications was also significantly correlated with a higher attitudes score on the gender roles and IPV index (Table 3).

**Table 3:** Mean score for attitudes towards gender roles and IPV index at baseline and at follow-up by sociodemographic characteristics and IPV status in Australia-born women and women born in conflict-affected countries.

	Australia-born women (n = 528)		Women born in conflict-affected countries (n = 583)	
Socio-demographic characteristics and IPV	Attitudes towards gender roles and IPV Index: Mean (SD)		Attitudes towards gender roles and IPV Index: Mean (SD)	
	Baseline	Follow-up	Baseline	Follow-up
**All**	0.2 (0.5)	0.2 (0.5)	0.7 (0.8)	0.7 (0.8)
**Age group (in years)**				
<25	0.4 (0.6)	0.3 (0.6)	0.9 (0.9)	0.7 (0.8)
25–34	0.2 (0.5)	0.2 (0.2)	0.7 (0.7)	0.7 (0.8)
35 and above	0.1 (0.3)	0.1 (0.4)	0.7 (0.8)	0.7 (0.8)
*p-values from F-test*	*p<0*.*001*	*p = 0*.*114*	*p = 0*.*048*	*p = 0*.*938*
**Highest level of educational attainment**				
No post-school qualification	0.3 (0.5)	0.2 (0.5)	0.9 (0.8)	0.8 (0.9)
Diploma or vocational education	0.2 (0.2)	0.2 (0.2)	0.6 (0.7)	0.6 (0.7)
University degree	0.2 (0.5)	0.2 (0.4)	0.6 (0.7)	0.6 (0.8)
*p-values from F-test*	*p = 0*.*052*	*p = 0*.*158*	*p<0*.*001*	*p<0*.*001*
**Employment status**				
Unemployed and others	0.3 (0.6)	0.3 (0.6)	0.8 (0.8)	0.8 (0.9)
Employed	0.2 (0.4)	0.1 (0.4)	0.5 (0.6)	0.5 (0.7)
*p-values from t-test*	*p = 0*.*002*	*p = 0*.*001*	*p<0*.*001*	*p<0*.*001*
**Intimate partner violence (IPV) at baseline**				
No IPV	0.2 (0.4)	0.1 (0.4)	0.6 (0.7)	0.6 (0.8)
Any IPV	0.5 (0.7)	0.4 (0.6)	0.9 (0.8)	0.8 (0.9)
*p-values from t-test*	*p<0*.*001*	*p<0*.*001*	*p<0*.*001*	*p = 0*.*001*
**Intimate partner violence (IPV) at follow-up**				
No IPV	0.1 (0.4)	0.1 (0.4)	0.6 (0.7)	0.6 (0.8)
Any IPV	0.5 (0.7)	0.3 (0.6)	0.9 (0.8)	0.8 (0.9)
*p-values from t-test*	*p<0*.*001*	*p<0*.*001*	*p<0*.*001*	*p = 0*.*001*

**Note:** Statistical tests *t* and *F* were conducted to examine the significant variation of mean score for attitudes towards gender roles and IPV index for each factor within the Australia-born women and women born in conflict-affected countries; *p*-values represent the significance level within the group.

### Intimate partner violence

The prevalence of any IPV was significantly higher in women born in conflict-affected countries compared to Australia-born women at both baseline interview and at follow-up interview (Tables 1 and 2). At baseline, 43.4% (95% CI 39.4–47.4) of women born in conflict-affected countries reported experiencing physical or psychological IPV, compared to 25.9% (95% CI 22.2–29.7) of Australia-born women. The prevalence of IPV reported at follow-up showed significant correlation with the prevalence of IPV reported at baseline for women born in conflict-affected countries (r = 0.52, *p*<0.001) and for women born in Australia (r = 0.46, *p*<0.001), respectively (Table 2).

Higher prevalence of any IPV was significantly associated with younger age, lower level of education, and unemployment in both Australia-born women and women born in conflict-affected countries at baseline and at follow-up. Higher attitudes score on the gender roles and IPV index was associated with greater prevalence of IPV in both Australia-born women and in women born in conflict-affected countries at baseline and at follow-up (*p*<0.001 in all cases). Women with the highest attitudes score on the gender roles and IPV index had the highest prevalence of IPV (Table 4). All the predictors found to be statistically significant (p <0 .05) in the bivariate analyses (Table 4) were included in the multiple logistic regression model.

**Table 4:** Results from bivariate analysis: prevalence of intimate partner violence (IPV) at baseline and at follow-up by sociodemographic characteristics, and by Attitudes towards gender roles and IPV index, for Australia-born women and women born in conflict-affected countries.

	Australia-born women (n = 528)						Women born in conflict-affected countries (n = 583)					
	Baseline			Follow-up			Baseline			Follow-up		
Sociodemographic characteristics and Attitudes towards gender roles and IPV index	Total Women	Prevalence of IPV		Total women	Prevalence of IPV		Total women	Prevalence of IPV		Total women	Prevalence of IPV	
	Number	No.	Row %	Number	No.	Row %	Number	No.	Row %	Number	No.	Row %
**All**	528	137	25.9	528	143	27.1	583	253	43.4	583	256	43.9
**Age groups (in years)**												
<25	115	48	41.7	115	53	46.1	102	57	55.9	102	58	56.9
25–34	321	73	22.7	321	75	23.4	362	146	40.3	362	147	40.6
35 and above	92	16	17.4	92	15	16.3	119	50	42.0	119	51	42.9
*Chi-square test*: *p values*			*p<0*.*001*			*p<0*.*001*			*p = 0*.*02*			*p = 0*.*01*
**Highest level of educational attainment**												
No post-school qualification	225	76	33.8	225	80	35.6	287	154	53.7	287	163	56.8
Diploma or vocational education	136	28	20.6	136	35	25.7	99	40	40.4	99	36	36.4
University degree	167	33	19.8	167	28	16.8	197	59	29.9	197	57	28.9
*Chi-square test*: *p values*			*p<0*.*01*			*p<0*.*001*			*p<0*.*001*			*p<0*.*001*
**Employment status**												
Unemployed	211	84	39.8	211	81	38.4	427	212	49.6	427	215	50.4
Employed	317	53	16.7	317	62	19.6	156	41	26.3	156	41	26.3
*Chi-square test*: *p values*			*p<0*.*01*			*p<0*.*001*			*p<0*.*001*			*p<0*.*001*
**Attitudes towards gender roles and IPV Index**												
0 (disagreement for all three domains)	421	87	20.7	439	107	24.4	253	90	35.6	284	108	38.0
1 (agreed in one domain)	92	39	42.4	77	26	33.8	243	102	42.0	204	93	45.6
2 (agreed in two domains)	14	10	71.4	10	9	90.0	73	52	71.2	73	41	56.2
3 (agreed in all three domains)	1	1	100.0	2	1	50.0	14	9	64.3	22	14	63.6
*Chi-square test*: *p values*			*p<0*.*001*			*p<0*.*001*			*p<0*.*001*			*p<0*.*001*

**Note:** Chi-square (χ2 test) was conducted to examine the significant variation of prevalence of IPV for each factor within the Australia-born women and women born in conflict-affected countries; *p*-values represent the significance level within the group.

#### Multiple logistic regression analysis

Adjusted odds ratios (aORs) from multiple logistic regression for both Australia-born women and women born in conflict-affected countries showed significant associations between attitudes towards gender roles and IPV and the prevalence of IPV at both baseline and follow-up interviews (Table 5). Women with the highest attitudes towards gender roles and IPV indices of 2 or 3 had the highest odds of IPV, followed by women with the attitudes towards gender roles and IPV index of 1, and the lowest odds of IPV in women with the lowest attitudes towards gender roles and IPV index of 0. Compared to women with an attitudes towards gender roles and IPV index of 0, Australia-born women who had high attitudes towards gender roles and IPV indices of 2 or 3 had an aOR of 7.12 for IPV at baseline (95% CI 2.12–23.92) and an aOR of 10.59 for IPV at follow-up (95% CI 2.21–50.75). Women born in conflict-affected countries who had high attitudes towards gender roles and IPV indices of 2 or 3 had an aOR of 3.18 for IPV at baseline (95% CI 1.85–5.47) and an aOR for IPV of 1.83 at follow-up (95% CI 1.11–3.01). For both Australia-born women and women born in conflict-affected countries, the sociodemographic status of being unemployed was associated with higher aORs for IPV at baseline and also at follow-up (aOR range 1.75–2.74). For Australia-born women, those aged under 25 years had a higher aOR for IPV at baseline (aOR 2.14, 95% CI 1.06–4.29) and also at follow-up (aOR 3.24, 95% CI 1.64–6.44). For women born in conflict-affected countries, lack of post-school qualifications had a higher aOR for IPV at baseline (aOR 1.88, 95% CI 1.23–2.86) and also at follow-up (aOR 2.41, 95% CI 1.59–3.67) (Table 5).

**Table 5:** Multiple logistic regression results of sociodemographic characteristics and attitudes towards gender roles and IPV index, associated with intimate partner violence (IPV) for Australia-born women and women born in conflict-affected countries at baseline and follow-up: Adjusted odds ratios (aOR) with 95% confidence interval (95% CI).

	Australia-born women (n = 528)		Women born in conflict-affected countries (n = 583)	
^a^Sociodemographic characteristics and Attitudes towards gender roles and IPV index	Baseline IPV^b^	Follow-up IPV^b^	Baseline IPV^b^	Follow-up IPV^b^
	aOR (95% CI)	aOR (95% CI)	aOR (95% CI)	aOR (95% CI)
**Age group (in years)**				
<25	2.14 (1.06–4.29)*	3.24 (1.64–6.44)**	1.30 (0.74–2.28)	1.34 (0.76–2.36)
25–34	1.20 (0.64–2.23)	1.40 (0.75–2.61)	0.99 (0.63–1.53)	0.95 (0.61–1.48)
35 and above (reference category)	1.00	1.00	1.00	1.00
**Highest level of educational attainment**				
No post-school qualification	1.05 (0.60–1.83)	1.64 (0.93–2.85)	1.88 (1.23–2.86)**	2.41 (1.59–3.67)**
Diploma or vocational education	0.79 (0.43–1.44)	1.42 (0.79–2.55)	1.38 (0.81–2.32)	1.26 (0.74–2.13)
University degree (reference category)	1.00	1.00	1.00	1.00
**Employment status**				
Unemployed	2.74 (1.73–4.33)*	1.75 (1.13–2.73)*	1.94 (1.25–3.00)**	1.90 (1.23–2.93)**
Employed (reference category)	1.00	1.00	1.00	1.00
**Attitudes towards gender roles and IPV index at baseline**				
0 (reference category)	1.00	NA	1.00	NA
1	2.34 (1.42–3.87)**		1.11 (0.76–1.62)	
2–3	7.12 (2.12–23.92)**		3.18 (1.85–5.47)**	
**Attitudes towards gender roles and IPV index at follow-up**				
0 (reference category)	NA	1.00	NA	1.00
1		1.40 (0.81–2.42)		1.17 (0.80–1.72)
2–3		10.59 (2.21–50.75)**		1.83 (1.11–3.01)**

**Notes.** *Adjusted odds ratios (aORs) are significant at p < 0.05

** aORs are significant at p < 0.01. NA, not applicable

^**a**^All the predictors included in the multiple logistic regression model for each community sample was found to be statistically significant (p < 0.05) in bivariate analysis (Table 4)

^**b**^The outcome variable IPV for multiple logistic regression model was coded as: 0 = no IPV and 1 = any IPV.

## Discussion

This study found that attitudes towards gender roles and IPV and the prevalence of IPV were significantly correlated in both women born in Australia and women born in conflict-affected countries. Compared to women born in Australia, women from conflict-affected countries in both pregnancy and in the post-natal period had significantly higher prevalence of IPV, and attitudes that more strongly endorsed gender roles and IPV. Irrespective of country of birth, women who were unemployed and those who most strongly endorsed attitudes supportive of gender roles and IPV were most likely to have experienced IPV.

A strength of this study is that it is the first to examine associations between IPV prevalence and attitudes towards gender roles and IPV in women from conflict-affected countries, with a host nation comparison. The study addresses a need for research on relationships between conflict-related populations and IPV [35]. To our knowledge, it is the first systematic study of associations between IPV and attitudes towards gender roles and IPV in conflict-affected displaced women attending antenatal clinics in a high-income resettlement country. As such, the study’s findings can inform clinical interactions and potential interventions for IPV in health settings of high-income countries where women are frequently seen at a critical point in their lifetime, and at the beginning of foetal and child health. The study is robust, having applied standardized instruments from the WHO Multi-Country Study on Women’s Health and Life Experiences Questionnaire to assess attitudes and IPV [31–32] to a systematic sample of 583 women from conflict-affected countries and 528 women born in Australia. Another strength of this study is that it uses a prospective cohort design with repeat assessment of women’s attitudes, experiences of IPV, and associations between attitudes and IPV over time.

A limitation of the study is that the analysis did not include an evaluation of causality or directionality of associations between attitudes towards gender roles and IPV and exposure to IPV. The sampling of women from antenatal clinics makes these findings of unknown generalizability to women who are not pregnant, but there is no reason to believe that the study’s general outcomes would be different in a sample of women who were not pregnant. IPV can be under-reported due to stigma [2, 4]. Under-reported IPV would make the associations found between attitudes towards gender roles and IPV and reported IPV to be underestimates and therefore would not negate the study findings. Not including sexual abuse by a partner in the IPV inquiries, based on advice by cultural experts, would also lead to an underestimate of associations between IPV and attitudes towards gender roles and IPV, and therefore would not negate the study’s findings. A limitation of the study is that the low number of Australia-born women who held attitudes strongly supportive of gender roles and of men’s use of IPV in some circumstances, led to wide confidence intervals for their aORs for IPV, although statistical significance was established. Due to the wide confidence intervals, it is important not to take at face value the high absolute values of the aORs for IPV in Australia-born women or to compare them with aORs for IPV in women from conflict-affected countries. Studies with larger numbers of host-nation women would be needed to confirm the trend for higher aORs for IPV statistically. A limitation of this study is that it does not interview partners for their attitudes or identify characteristics of partners which could provide insight into protective or risk factors regarding perpetrating IPV, such as income differentials between a woman and her partner [36], alcohol use by partners [29], or attitudes of partners or other family members towards gender roles and IPV [13, 37–39].

Traditional societies can endorse gendered roles and attitudes supportive of IPV more strongly, and this study adds to others suggesting that such culturally informed attitudes may be transferred to the country of settlement [25, 37–39]. That woman affected by IPV may paradoxically hold attitudes that support men’s use of IPV may be explained by a lifetime experience of, and personal integration of, traditionally held gendered attitudes [4, 37, 40]. Abused women may also hold gender role attitudes supportive of IPV because of a commonly experienced self-blame or low self-worth, which can contribute to excusing IPV [4, 12, 37, 40, 41]. Gender role attitudes supportive of IPV may be adopted as a ‘coping mechanism’ by women being subjected to IPV [23]. Abused women may be more prone to rationalizing IPV when they are materially or socially dependent upon their partners, a significant problem for women from conflict-affected countries who are often isolated and on low incomes during settlement [39, 42, 43].

Women from conflict-affected countries can face greater barriers to leaving violent partnerships in high-income countries of resettlement, even in cases where there are women-protective laws and resources [8, 9, 37, 39, 41, 44]. This may contribute to higher rates of IPV among women from conflict-affected countries compared to host-nation women. Women from backgrounds where personal insecurity and fear of authorities predominate, may refrain from reporting IPV in a host country due to actual or feared harm, discrimination and racism. After 9/11, a study found that most Arab immigrant women in the United States no longer felt that they would contact police or legal services about IPV, due to fears that their husbands would be deported or treated unfairly by the legal system [41]. Women who have lived through armed conflict may be more likely to excuse their partner’s violence due to knowledge of their partner’s trauma and their shared experience of contending with political violence and the challenges of displacement.

This study found that for both Australia-born women and women born in conflict-affected countries, being unemployed was associated with higher aORs for IPV. For Australia-born women, being younger was associated with a higher aOR for IPV. For women born in conflict-affected countries, lower education was associated with higher aORs for IPV. Younger age has been found to be a risk factor for IPV in countries where social attitudes more strongly support wife-beating, whereas education has been found to be a protective factor in countries where social attitudes more strongly support wife-beating [14].

Armed conflict and political violence are societal factors that have been associated with attitudes that condone IPV. In the 49-nation Demographic and Health Survey analysis, men and women living in conflict-affected countries were more likely to condone wife-beating [45]. Women who live in conflict-affected settings or who are displaced by armed conflict have been found to experience higher rates of IPV [9, 18–21, 24]. It has been proposed that existing levels of IPV may be exacerbated by exposure to conflict-associated violence, trauma, job loss by partners, male partners’ experience of torture, loss of social support, altered gender roles, and higher levels of stress [18, 45–47]. IPV prevalence and the mental health impacts of it can persist for years after conflict ends, and there is a need for targeted IPV interventions to assist affected women and families in optimal recovery from prior trauma to aide successful resettlement [20, 21, 24, 46, 47].

In this study, women born in Australia and women from conflict-affected counties showed no significant change between the period of pregnancy and follow-up in the postnatal period in their attitudes towards gender roles and IPV, in the prevalence of IPV by their last or current partner within the past 12 months, or in the strength of association between attitudes and IPV. The lack of change over time is particularly interesting as decreased endorsement of attitudes towards gender roles and IPV might be expected due to some level of acculturation to host-nation norms, and the existence of legal and material resources for women experiencing IPV [48]. This study’s finding of no change in attitudes is consistent with a study of Libyan Arab immigrants to the United Kingdom (UK), in which length of stay in the UK was not correlated with attitudes towards IPV [38]. Similarly, Bosnian refugee women living in the United States had attitudes towards wife-beating that were no different from those of non-refugee Bosnian women living in Bosnia-Herzegovina, despite prolonged residency in the United States that averaged 8 years [25]. An important policy and prevention consideration is to counter strongly held familial, cultural, and local social beliefs carried by women in conflict affected settlement communities about IPV and gender equality [25, 37–39]. There is, nevertheless, a need for cultural interpretation of attitudes that may appear to condone IPV. For attitudes to change, greater understanding is needed of the complex interplay of attitudes and beliefs that influence how women from non-Western traditions might interpret questions related to men’s entitlements and expectations. Further, by including many cultures in one analysis of women from conflict-affected countries, some features of non-Western societies may be overlooked which protect women from violence. Islam, for example, supports harmonious relationships and rejects violence against women in marriage [49].

IPV interventions amongst pregnant women who arrive in high-income countries from conflict-affected settings may, according to this study, benefit from interventions that target or reflect a sound understanding of possible endorsement of gender role attitudes and attitudes that appear to condone IPV [13, 26]. This study underscores why interventions urgently need to acknowledge the importance of changing attitudes towards gender roles and IPV amongst women [2, 15, 17, 41, 50]. Perinatal clinics offer an important opportunity for carrying out sensitive screening for IPV and offering carefully adapted interventions for IPV among women from conflict-affected countries in addition to women from the host population. Prevention of IPV among women who have experienced armed conflict prior to arrival in a high-income country requires sociocultural sensitivity and attention to socioeconomic factors, which are essential in the process of challenging gender values and attitudes that support IPV [2, 17, 38, 41].

Regarding interventions, the findings of this study suggest that in Australian antenatal clinics, and possibly those in other high-income countries, a background of armed conflict or political violence may indicate a risk factor for being at higher risk of IPV. The findings also suggest that in perinatal clinics, clinicians should screen for, and respond to, IPV in a manner that is sensitive to women’s attitudes towards her role within the spousal/partner relationship and how women may rationalise violence perpetrated by an intimate partner. Practitioner interactions with conflict-affected need to be culturally specific enough to appreciate women’s attitudes towards gender roles and the association with men’s use of violence [17, 37, 38, 41, 44].

## Conclusion

To our knowledge this is the first systematic study to examine the prevalence of IPV amongst women from conflict-affected countries and the association between women’s attitudes to gender roles and men’s use of IPV. The comparison group of Australian born women, and two timepoints across the critically important periods of pregnancy and postpartum, add considerably to the study. The need to understand the aetiology of IPV requires a better understanding of attitudes towards it, and the contextual factors including adherence to norms that tolerate or condone gender unequal roles and IPV. There are few large and robust studies that can contribute to policy and practice in this field, and our study addresses this gap, including both women born in the host country and those who come to settle from countries impacted by conflict.

## Supporting information

S1 Data (XLSX)
